# Gene and Genome Duplication in Spiders

**DOI:** 10.1002/jez.b.23304

**Published:** 2025-05-25

**Authors:** Chetan Munegowda, Matthias Pechmann, Nikola‐Michael Prpic‐Schäper, Natascha Turetzek

**Affiliations:** ^1^ AG Zoologie mit dem Schwerpunkt Molekulare Entwicklungsbiologie, Institut für Allgemeine Zoologie und Entwicklungsbiologie Justus‐Liebig‐Universität Gießen, Carl‐Vogt‐Haus Gießen Germany; ^2^ Institute for Zoology, Department of Developmental Biology University of Cologne Cologne Germany; ^3^ Evolutionary Ecology, Faculty of Biology, Biocenter Ludwig‐Maximilians‐University of Munich Planegg‐Martinsried Germany

**Keywords:** neofunctionalization, spiders, usurpation, whole genome duplication (WGD)

## Abstract

Gene and genome duplications are widely observed across various organisms, including plants, yeasts, and animals. Numerous studies link gene duplications to the emergence of novel phenotypes, supporting the hypothesis that duplication events are advantageous for adaptive evolution. Whole‐genome duplications (WGD) are especially prevalent in plants and have also occurred ancestrally in vertebrates. However, large‐scale duplication events in other animal groups remain understudied, partly due to limited genomic resources. Arthropods, particularly insects, represent one of the most diverse animal clades in terms of both species and phenotypic diversity. With increasing availability of chromosome‐level genomes, large‐scale duplications appear to be rare in insects but are more frequent in chelicerates (e.g. spiders, scorpions, and horseshoe crabs). This makes chelicerates an intriguing group for comparing the mechanisms, fates, and evolutionary impacts of large‐scale duplications with those seen in plants and vertebrates. In this review, we synthesize and discuss current research on WGD in spiders and discuss different scenarios for genes following gene duplication events (conservation, nonfunctionalization, subfunctionalization, specialization, drift, neofunctionalization) in the context of experimental studies. We hypothesize if there might be common trajectories after duplication and how these could be tested.

## Graphical abstract

In chelicerates whole genome duplications (WGDs) were identified in distinct groups. While there is evidence that Xiphosurans/horseshoe crabs had three rounds of WGD and arachnopulmonates (e.g. spiders and scorpions) had one WGD, in many other arachnid groups no WGD was identified. The origin of the WGD events in chelicerates is not fully understood. Similarly, although possible fates after gene duplication, e.g. subfunctionalisation, nonfunctionalisation, or neofunctionalization are hypothesized in the literature, a systematic approach to reveal how many of the duplicated genes after the chelicerate WGDs evolved which fate is still missing. The increasing availability of high‐quality chromosome‐level genomes and comprehensive comparison of gene evolution represent a unique opportunity to study WGDs, gene duplication evolution, and diversification in chelicerates in comparison to findings from plants or vertebrates. Cumulative number of spider genomes sequenced by year as reported on NCBI accessed on 10th April 2025, except for *Parasteatoda tepidariorum* published 2017 by Schwager et al. Please note blue line includes the chromosome‐level genomes and the *Argiope bruennichi* genome in 2020 was the first chromosome‐level spider genome.

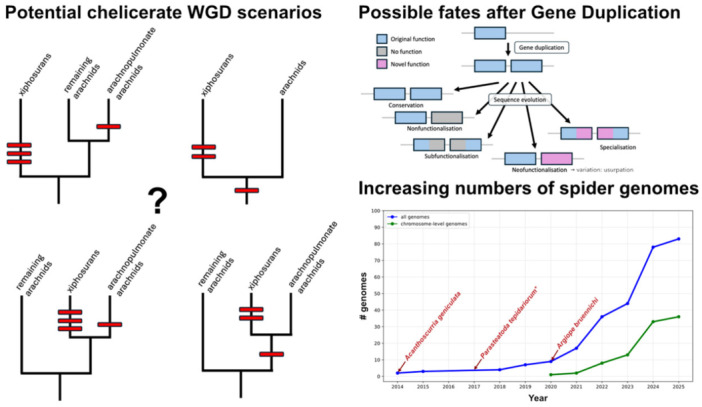

## 1 Introduction

All organisms continuously adapt their phenotype to their environment and evolve, for example, new morphologies, behaviors, and physiological functions. Genetic changes are the basis for such adaptive capabilities. One way of providing the genetic material for novel phenotypes is the origin of new genes. For a long time the origin of new genes simply from previously non‐transcribed, suddenly and randomly transcribed genetic material has been regarded as a rare process (Jacob 1977; Tautz 2014). In recent years, however, such de novo genes (Van Oss and Carvunis 2019; Tautz 2014; Tautz and Domazet‐Lošo 2011) have been identified in many taxa (An et al. 2000; Baalsrud et al. 2018; Chen et al. 2014; Heinen et al. 2009; Li et al. 2010; Weisman 2022) and shown to be a significant source of genetic functions for adaptation (Bornberg‐Bauer et al. 2015; Neme et al. 2017; Parikh et al. 2022).

**Figure 1:**
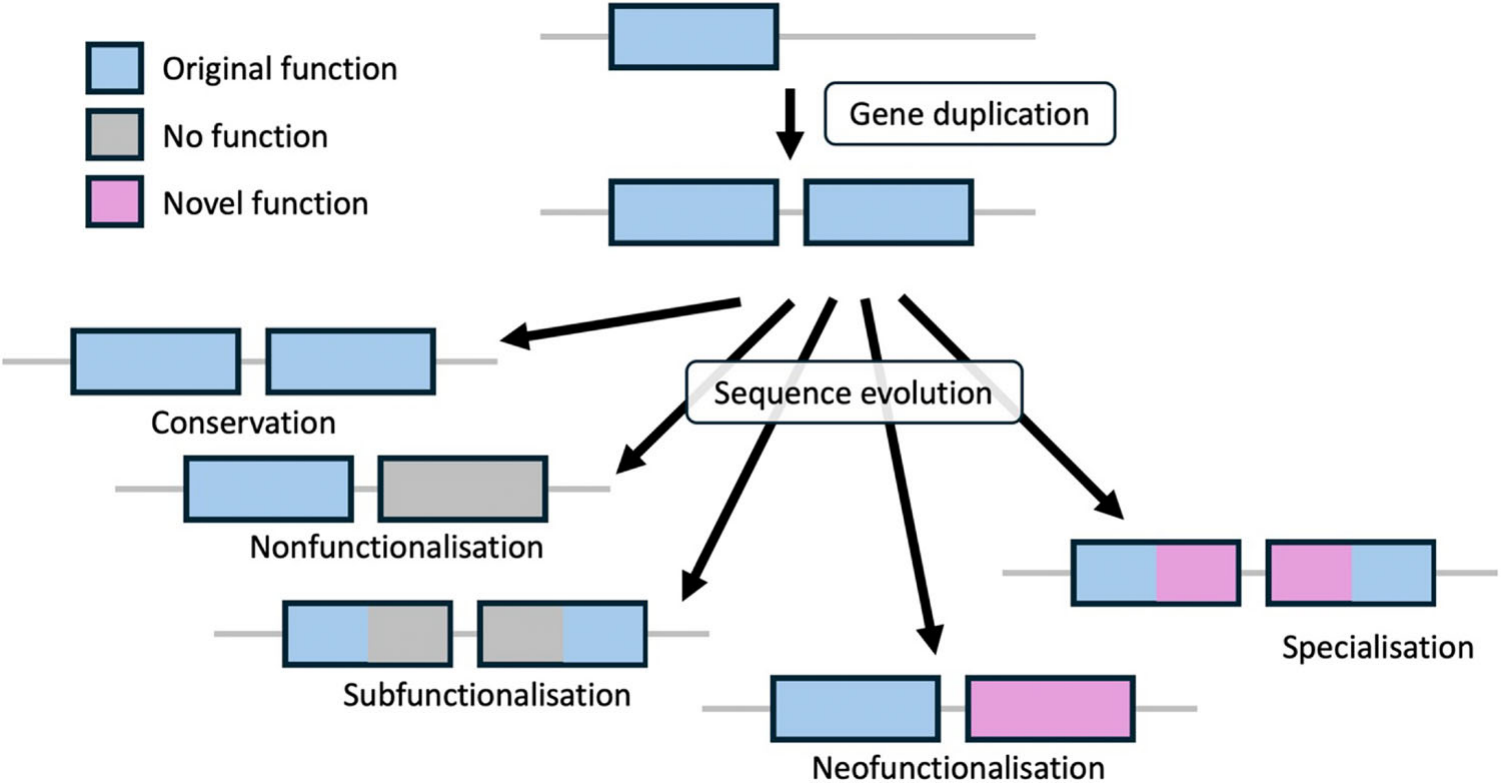
Current categorization of possible fates of gene pairs after duplication. Either both copies are retained in the genome (conservation), or sequence evolution alters one or both copies to restore the pre‐duplication status. Restoration of the pre‐duplication status may be the only outcome of sequence evolution (nonfunctionalisation, subfunctionalisation) or sequence evolution may additionally result in novel gene functions (neofunctionalisation, specialisation) (After DeGiorgio and Assis 2021).

Another way to obtain new gene functions for novel phenotypic features are changes in the regulation of existing genes. In this way, tried and tested gene products can be reused to generate novel phenotypic features. Indeed, regulatory changes are currently believed to be the main driver of phenotypic diversification (Carroll 2008, 2005; Carroll et al. 2008; Sommer‐Trembo et al. 2024). However, altering the regulation of an existing gene always holds the risk of losing the previous role of the gene. In addition, most of these examples were based on comparisons of closely related species with comparatively little variation concerning their protein coding genes. It has therefore been suggested that gene duplications could be another major driver of phenotypic evolution (Ohno 1970). Gene duplication provides the advantages, while compensating for the disadvantages: one gene copy ensures that the original gene function remains untouched, the second gene copy can be changed for a new phenotypic feature. Major “explosions” of phenotypic diversity could therefore be correlated with “bursts” of gene duplications, especially duplications of entire genomes (whole‐genome duplications, WGD) (e.g. Amores et al. 1998; Glasauer and Neuhauss 2014; Soltis and Soltis 2016; Van de Peer et al. 2009; Walden et al. 2020). Apart from the initial problem of dose compensation after the WGD event, such polyploid organisms possess “spare” copies of each single gene of their genome and should therefore possess a full range of genetic material for rapid and adaptive evolutionary change (e.g. Crow 2006; Flagel and Wendel 2009; Kondrashov 2012; Pegueroles et al. 2013; Qian and Zhang 2014). The evolutionary trajectories of gene pairs after gene duplication are diverse and have been classed into five categories: conservation, nonfunctionalisation, subfunctionalisation, neofunctionalisation and specialisation (Figure 1) (Summarized in DeGiorgio and Assis 2021; Force et al. 1999; Lynch and Force 2000; Ohno 1970; Prince and Pickett 2002).

While gene duplication therefore might represent a prime mechanism for the evolution of phenotypic novelty in theory, in practice it is difficult to envision how gene duplication takes place in an organismal setting. Especially in multicellular animals (Metazoa), the duplication of a gene is often harmful to the individual, let alone the duplication of chromosomes or the duplication of an entire genome (Katju and Bergthorsson 2013). WGD are therefore rare events in animal evolution, but if they do occur, they should correlate with a major increase of phenotypic novelties, if the idea of a link between gene duplications and phenotypic evolution is correct. One striking example linking gene or even genome duplication and with significant phenotypic key innovations is the evolution of venom across diverse metazoan lineages (e.g. Farhat et al. 2023; Hargreaves et al. 2014; Wong and Belov 2012; Zancolli and Casewell 2020). Therefore, WGD are highly sought‐after and intensively studied when identified.

Arthropods, especially insects, are astonishing in terms of their species richness and phenotypic diversification. Although signals for insect large‐scale duplication events in genomes were found (Driscoll et al. 2020; Duan et al. 2020; Li et al. 2018), WGDs in insects seem to be rare (Lokki and Saura 1980; Roelofs et al. 2020). Beginning with the comparative study of Hox genes, major regulators of the body plan (Damen et al. 1998), several developmental genes have been studied in spiders over the past 30 years. Surprisingly, it was repeatedly observed that isolating fragments of spider homologues of developmental genes, known from *Drosophila melanogaster* (fruit fly) embryonic development, resulted in two very similar copies in spiders (Abzhanov et al. 2001; Damen et al. 2002, 1998; Janssen et al. 2015, 2010, 2008; Pechmann et al. 2015; Prpic et al. 2003; Schomburg et al. 2015; Schwager et al. 2007; Stollewerk et al. 2003). No explanation for this phenomenon was available until 10 years ago, when the analysis of the first spider genomes suggested that the genome of spiders might have undergone a WGD (Clarke et al. 2015; Schwager et al. 2017). Interestingly, spiders also display large variations in genome size and chromosome numbers (e.g. Datta and Chatterjee 1989; Král et al. 2019; Kumbıçak et al. 2014; You et al. 2024), and some species are reported to have up to 47 chromosomes (*Macrothele cretica*: NCBI Assembly: GCA_964417605.1) or around 9 Gb genome size (*Maratus speciosus*: NCBI Assembly: GCA_963932465.1). A direct link of large spider genomes and chromosome number with the WGD is, however, difficult. There are also many occasions of genome size variation or expansion in insects without an accompanied WGD, most likely explained by chromosome fusion and division (e.g. in crickets Kataoka et al. 2022). In addition, a recent comparative chromosome number study including more than 15,000 species across the tree of life revealed that in plants polyploidy and changes in chromosome number and speciation are much stronger associated than in animals (Román‐Palacios et al. 2021). Discussing a link between genome size expansion, chromosome number and WGD and the underlying mechanisms, although interesting, is beyond the scope of this review and seems to be a future avenue for large‐scale genome comparisons especially with more and more high‐quality genome sequences becoming available.

In this respect, spiders are an exciting group: They not only show a great variation in their genomes, but also exhibit numerous unique adaptations ranging from morphological and behavioural to developmental and physiological phenotypes. Thus, their striking phenotypic diversity might be linked to the postulated WGD, in this way making them a unique model for the study of WGD, genome evolution, and phenotypic evolution in metazoan animals.

## 2 Genome Duplication in Xiphosurans

Spiders (Araneae) belong to a larger group of arthropods, termed chelicerates (Chelicerata). Apart from spiders, the Chelicerata comprise groups like mites, harvestmen, sun spiders and scorpions. Also, the marine horseshoe crabs (Xiphosura) belong to the chelicerates, despite their name suggesting that they are a species of “crab”. The genomes of xiphosurans contain even more duplicated genes than the genomes of spiders. Some genes are present in 6 or more copies in xiphosurans, and it has therefore been suggested that the genome of extant xiphosurans has undergone not only one WGD, but two or three rounds of WGD (Gong et al. 2019; Kenny et al. 2016; Nong et al. 2021; Shingate et al. 2020; Simpson et al. 2017; Zhou et al. 2020).

**Figure 2:**
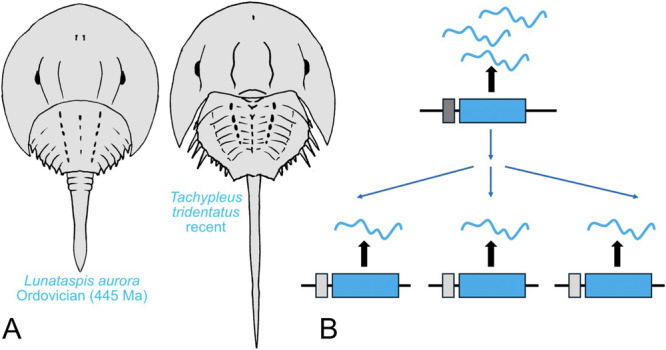
Morphological stasis in the Xiphosura despite several WGD events. (A) The external features of the body plan of the xiphosurans have remained almost unchanged during their phylogeny as exemplified by a fossil representative (*Lunataspis aurora*) from the Ordovician period (445 million years ago (Rudkin et al. 2008)) and a recent species (*Tachypleus tridentatus*). (B) Hypothetical mitigation of WGD in xiphosurans. The regulatory region (dark grey) of the original gene (top) drives a certain amount of gene product (symbolised by the blue wavy lines). After multiplication of the original gene by WGD (blue arrows) the action of the regulatory region of all gene copies (bottom) is reduced to an appropriate fraction of the original action (light grey), the final amount of gene product, however, remains the same. This hypothetical process represents a type of subfunctionalisation (dosage subfunctionalisation (Gout and Lynch 2015)).

As already mentioned above, WGD in animals are rare events and multiple rounds of WGD are extremely scarce. This makes xiphosuran genomes a prime target for the study of gene duplication mechanisms. However, the case of the rampant gene duplication in xiphosurans at the same time poses a problem for the current ideas of genome evolution by genome duplication (see also discussion in (Sharma 2023)). First, although in principle WGD provide new genes, a frequent outcome after gene duplication is believed to be the disuse of one copy (nonfunctionalisation) (Ohno 1970; Prince and Pickett 2002), to avoid deleterious effects of the doubled gene activity. It is expected that even after a WGD most duplicates quickly become nonfunctionalised, deteriorate into pseudogenes and are finally lost again from the genome. By contrast, however, nonfunctionalisation does not appear to be a major fate of duplicated genes in the Xiphosura, because many genes are present in several apparently well‐conserved copies. Second, WGD is thought of as a facilitator of phenotypic diversification and should therefore lead to many phenotypic novelties and an increase in species numbers (Amores et al. 1998; Glasauer and Neuhauss 2014; Soltis and Soltis 2016; Van de Peer et al. 2009; Walden et al. 2020). Both effects are not observed in the Xiphosura: based on their fossil record, at least their external morphology has remained almost unchanged since the Ordovician period (Figure 2A), and with only four extant species, the Xiphosura are among the least species‐rich groups of the Chelicerata. Interestingly, vertebrates and many plant groups have ancient WGDs, but not all WGD events necessarily resulted in species or morphological diversification. In plants with ancient WGDs species rich crown groups are often accompanied by species‐poor sister clades (Schranz et al. 2012) and while the vertebrate WGD led to morphological diversification in gnathostomes this is not true for cyclostomes (Yu et al. 2024). To explain this, many studies assumed that a WGD alone might not be sufficient to cause diversification, but more complex explanations including ecological opportunities and changing environmental conditions are required (Schranz et al. 2012; Santini et al. 2009).

It remains the question, why there are so many duplicated genes retained in the xiphosuran genome? Three events of WGD are currently postulated, and the latest of them appears to be relatively recent within the lineage of the extant species (Nong et al. 2021; Roelofs et al. 2020; Shingate et al. 2020), while the other WGD are older, but there is no consensus about their exact age. It appears, therefore, as if xiphosurans underwent repeated rounds of WGD at different timepoints during their phylogeny, but were not affected by them in any obvious way. Is there a hitherto undescribed mechanism in xiphosurans to mitigate or entirely block the effects of large‐scale gene duplications? For example, xiphosurans may extensively utilize subfunctionalisation of the regulatory regions of the duplicates (Figure 2B) to restore the pre‐duplication state. This would prevent selection against further WGD and thus could explain why WGD occur repeatedly in xiphosurans. The distribution of the original gene expression level among functionally complementary regulatory regions could additionally explain why so many duplicated genes are still present in the genome: they are all still necessary to produce the original amount of gene product (Force et al. 1999; Lynch and Force 2000) and therefore may not undergo nonfunctionalisation (dosage subfunctionalisation (Gout and Lynch 2015)). One experimental support for potential dosage subfunctionalisation was found for the duplicates of the Hox genes *labial* and *Deformed* which show largely overlapping spatial and temporal expression during embryonic development (Gainett et al. 2023). Finally, a strong focus on subfunctionalisation would marginalise processes like neofunctionalisation, which could explain why WGD in xiphosurans does not lead to phenotypic diversity. Therefore, it would be very interesting to systematically study the function of xiphosuran gene duplicates: are all copies expressed, is there pervasive evolution of the regulatory regions of gene duplicates, and what role(s) do the individual gene copies have?

## 3 Pervasive Spider Gene Duplicates: Lineage‐Specific WGD or Shadow of an Older WGD?

Initial analyses of spider transcriptome and genome sequences indicated that there has been a WGD event in the phylogenetic lineage leading to the spiders (Clarke et al. 2015; Schwager et al. 2017). However, the timepoint of this event has been difficult to pinpoint exactly. The analysis by Clarke et al. 2015 suggested that the WGD event occurred relatively late in arachnid evolution. The analysis indicated a timepoint after the split between spiders and scorpions, probably as late as shortly before the radiation of the higher spiders (Araneomorphae). The origin and further radiation of the araneomorph spiders is generally believed to have been fuelled primarily by their web building behaviour, diverse silk glands and silk types (Alicea‐Serrano et al. 2024; Blackledge et al. 2009; Bond et al. 2014; Bond and Opell 1998; Craig 2003; Vollrath and Selden 2007). Therefore, Clarke et al. (2015) proposed for this “WGD late” scenario that the WGD may have provided the novel gene functions required for the evolutionary diversification of the silk producing apparatus. By contrast, the analysis by Schwager et al. (2017) indicated that the WGD event occurred early in arachnid evolution, well before the divergence of spiders and scorpions. In this interpretation, the WGD event defines a monophyletic group comprising scorpions and spiders that has subsequently been named as Arachnopulmonata (Sharma et al. 2014). The evolutionary significance of this “WGD early” scenario, however, is less clear and cannot be linked in a straightforward way to specific arachnopulmonate novelties, although one could hypothesize a contribution to key innovations like the arachnopulmonate book lungs and changes in reproductive behaviour (discussed in Ontano et al. 2021). Currently the concept of the Arachnopulmonata (Sharma et al. 2014) comprises not only the scorpions and spiders (Araneae), but also pseudoscorpions, whip scorpions (Urogypi), sprickets (Schizomida) and whip spiders (Amblypygi). Fittingly, the chromosome‐level genomes for a whip spider and a whip scorpion also support a WGD (Gainett and Sharma 2020; Kulkarni et al. 2024), and many duplicated developmental genes and miRNAs were identified in a pseudoscorpion (Ontano et al. 2021) thus supporting the “WGD early” scenario in Arachnopulmonata (Kulkarni et al. 2024; reviewed in Sharma and Gavish‐Regev 2024). The WGD event at the base of such a diverse group might therefore be viewed as a “general basis for species and phenotypic diversification” by providing many new genes. All studies agreed that the WGD event detected in arachnopulmonates, is independent of the WGD events detected in the genomes of extant horseshoe crabs. This is further supported by a current chelicerate phylogeny (Ballesteros et al. 2022), the lack of a WGD signal or duplicated gene clusters in sea spider (Pycnogonida) (Papadopoulos et al. 2024) and several non‐arachnopulmonate species like harvestmen (Gainett et al. 2021), mites and ticks (Acariformes and Parasitiformes) (studied e.g. in Aase‐Remedios et al. 2023; Kulkarni et al. 2024; Ontano et al. 2021), and sun spiders (Solifugae) (Gainett et al. 2024).

However, the evidence for a WGD in spiders was weak in the earlier studies, and other early studies of spider genomes were not able to identify a WGD in the genome sequence of several spider species (Babb et al. 2017; Sanggaard et al. 2014). Subsequent studies were either tentatively supportive, attributing the high number of duplicated genes in spiders either to WGD or to tandem gene duplication (Cerca et al. 2021; Fan et al. 2021; Miles et al. 2024; Schöneberg et al. 2024), or were unable to support the presence of a WGD in spiders (Sheffer et al. 2021). More recently, however, it has been argued that the detection of WGD in spiders is not possible with the currently available methods and data (Thomas et al. 2024). Thomas et al. 2024 propose that the current support for the presence of WGD in arachnopulmonates as well as the absence of WGD in non‐arachnopulmonate arachnids might both be artefacts. Only the WGDs in horseshoe crabs are robust and are strongly supported in their analysis. Although this study represents a promising approach to study WGD in chelicerates, including more species with and without WGD like harvestmen, pseudoscorpions, sea spiders, sun spiders and whip scorpions would provide a more comprehensive picture. Especially since experimental evidence supporting the WGD event in Arachnopulmonata, based on either whole genomes or specific gene families are accumulating (see examples below and citations above). For the homeobox gene family of transcription factors a pervasive duplication in arachnopulmonates was identified ranging between 50% and 60% compared to below 24% in other arthropods (Leite et al. 2018). An analysis of the clustering of these duplicated homeobox proteins revealed that the majority of these paralogs were dispersed and not the result of tandem duplications (Leite et al. 2018). This was further confirmed by a recent study analysing eight chromosome‐level spider genomes, where in addition to the Hox genes, most other homeobox gene clusters were also found in two copies (Aase‐Remedios et al. 2023). These clusters were even more conserved in terms of gene content than the vertebrate homeobox clusters (Aase‐Remedios et al. 2023). Thomas et al. 2024, however, suggest that previous evidence for a WGD may have been misinterpreted due to a narrow focus on particular genomic regions (specifically the homeobox family genes and Hox genes in particular). This perspective appears to overlook a range of studies that have examined other gene families and developmental pathways, including the Wnt genes (Harper et al. 2021; Janssen et al. 2021), the Sox genes (Baudouin‐Gonzalez et al. 2021; Bonatto Paese et al. 2018; Paese et al. 2018), the Fox genes (Janssen et al. 2022; Schomburg et al. 2022), or eye development (e.g. Baudouin Gonzalez et al. 2025; Baudouin‐Gonzalez et al. 2022; Janeschik et al. 2022a; Medina‑Jiménez et al. 2024; Propistsova et al. 2025; Samadi et al. 2015; Schomburg et al. 2015) (for a broader discussion see also (Kulkarni et al. 2024)). Beyond gene families, there is additional evidence from noncoding RNAs, microRNAs, which show high duplication rates in spiders, a scorpion, a horseshoe crab (Leite et al. 2016) and a pseudoscorpion (Ontano et al. 2021), but not in other chelicerates and arthropods. These studies are further supported by several more recently sequenced chelicerate genomes, which in addition to finding duplicated clusters for several gene families (see references above) also show macrosynteny (Kulkarni et al. 2024) in arachnopulmonates (e.g. whip scorpion, pseudoscorpion, whip spiders) lacking in other chelicerate lineages (see references above) as well as in the sea spider (Papadopoulos et al. 2024).

This raises the question of why the arachnopulmonate WGD, which has been strongly supported by numerous studies, could not be confirmed by Thomas et al. 2024. This discrepancy could likely be attributed to the relatively long evolutionary divergence since the WGD event, which is among the oldest known in animals, leading to the introduction of substantial noise. In combination with strict criteria for gene synteny identification, conventional WGD detection methods may be affected (for a more detailed discussion please see: Kulkarni et al. 2024).

In conclusion, although numerous studies continue to provide increasing evidence, the presence of a WGD in spiders is not set in stone, and the analysis remains complicated due to the long divergence time since the potential arachnopulmonate WGD event. If it is true that the current available methods and data basis are insufficient to unequivocally identify a WGD or to rule it out conclusively in non‐xiphosuran chelicerates (including spiders), then the following idea becomes a valid hypothesis: There has never been a WGD in spiders or the other non‐xiphosurans, and the high number of gene duplicates actually observed in spiders and the other groups must have originated through one or several other mechanisms, maybe even in lineage specific manners. One of these other mechanisms could be tandem duplication of genes. The evidence from several gene families to occur as duplicated synteny blocks and the conspicuously large number of duplicated genes observed in arachnopulmonates, however, suggests that tandem duplication events alone are unlikely to explain all duplications. Therefore, additional, possibly novel gene duplication mechanisms could be at work, that remain to be discovered.

**Figure 3:**
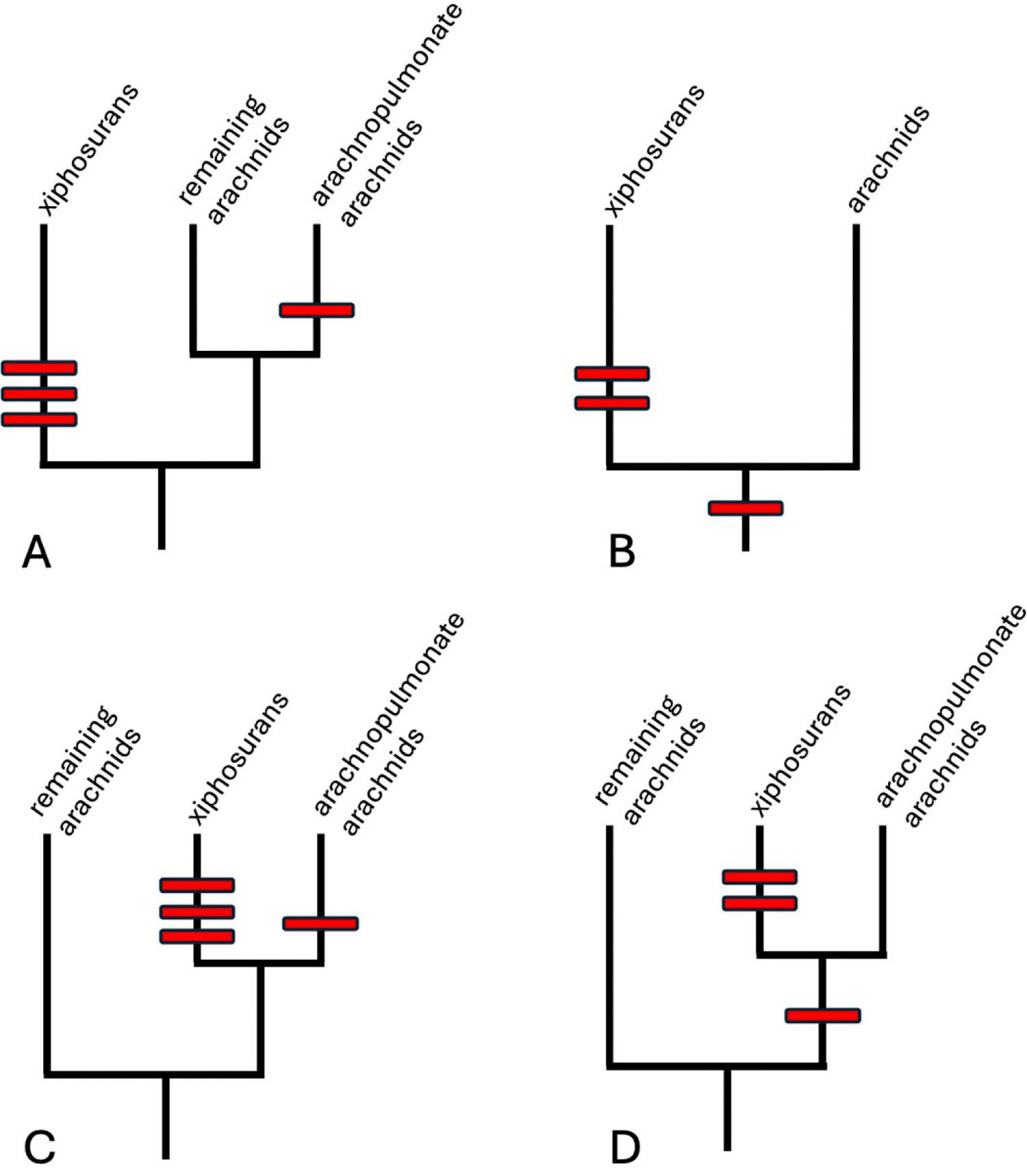
Alternative scenarios for the timing of possible WGD events in chelicerates. (A) Current majority view. The three postulated WGD events in the xiphosuran lineage are unrelated to the postulated WGD event that characterises the arachnid subbranch of the arachnopulmonates. (B) If the WGD in arachnopulmonate arachnids and the lack of a WGD in non‐arachnopulmonate arachnids are both artefacts of the current methods and data basis, then it is also possible that (at least) one of the WGD has occurred at the base of all chelicerates. No WGD has then to be postulated in the arachnid branch: if duplicated genes are present in certain lineages (e.g. spiders) then they trace from the ancestral chelicerate WGD, otherwise they have been eliminated from the genome. (C) Scenario that combines the current view that there have been independent WGD in xiphosurans and arachnopulmonates with recent phylogenetic results that xiphosurans are members of the arachnids and are the sister group to the arachnopulmonates. (D) If the scenario in C is combined with the notion that there has been no WGD within the arachnopulmonate lineage (scenario in B), then (at least) one of the WGD in the scenario in C can have occurred at the basis of the group combining xiphosurans and arachnopulmonates. The many duplicated genes in some arachnopulmonates then trace from this ancestral WGD, rather than from a separate WGD in the arachnopulmonates.

Alternatively, a causal link between the well‐supported WGD in xiphosurans and the occurrence of many duplicated genes in spiders could have been ruled out prematurely (Figure 3). Given the prevailing concept of the Xiphosura as the sister group of the remaining chelicerates (Arachnida) (Lozano‐Fernandez et al. 2019; Regier et al. 2010; Shultz 2007; Shultz 1990; Snodgrass 1938; Weygoldt and Paulus 1979) the oldest WGD may have occurred at the base of the Chelicerata. The WGD then subsequently might have been conserved to different degrees in xiphosurans and arachnids (Figure 3B). A weak conservation of the duplicated genetic material in arachnids would help explain the difficulty to reliably detect the traces of the ancestral WGD today. Even if the position of the Xiphosura as the sister group to the Arachnida is rejected and arachnids are not supported as monophyletic group (Ballesteros et al. 2022; Ballesteros and Sharma 2019; and reviewed in Sharma and Gavish‐Regev 2024), the Xiphosura branch close to the Arachnopulmonata and the observed gene duplicates in spiders could still trace from one single ancient WGD in the common ancestor of Xiphosura and Arachnopulmonata (Figure 3D).

## 4 Conservation: Are There Many “Identical Twin Genes” in Spider Genomes?

Immediately after gene duplication, the cell is confronted with a doubling of gene product amounts. The fastest way for the cells to deal with this situation is to adopt the new level of gene product amounts as being better than (or at least: not worse than) the previous amount (e.g. Kuzmin et al. 2020). This would lead to the retention of both copies as “identical twins” (conserved copies) in the genome. However, because the expression of most genes is meticulously regulated in the animal cell, it is generally not expected that gene conservation is a major fate of genes after large‐scale gene duplication and especially not after WGD.

The identification of conserved copies of a gene after gene duplication or WGD based on genome sequence analysis is theoretically straightforward. The original gene and its copy should retain the same regulation and function, and their nucleotide sequences should therefore be very similar, ideally including not only the coding sequence, but also their regulatory sequences. Indeed, examples of such conserved gene duplications have been identified in spiders, by specifically looking for genes where the logic behind gene conservation after duplication (i.e. “the more gene product the better”) makes immediate sense. Multiple copies of genes have been found for silk genes (Babb et al. 2017; Clarke et al. 2015; Sanggaard et al. 2014) (“more genes, more silk”) and for venom genes (Gendreau et al. 2017; Haney et al. 2016; Sanggaard et al. 2014) (“more genes, more venom”). However, these selective approaches suggest that the process of conservation after gene duplication leads to larger groups of highly similar genes, but not to pairs of duplicates, although the latter is what is indeed observed in most of the duplicated genes in spiders. Additionally, many examples of silk genes and venom genes are both attributed to repeated tandem duplications, not WGD (Babb et al. 2017; Gendreau et al. 2017; Miles et al. 2024; Sanggaard et al. 2014). It is therefore currently unclear, whether pairs of highly similar genes result from WGD. So far, the analysis of spider genome sequences has neither revealed pairs of genes that show almost identical nucleotide sequences, nor gene pairs where the similarities of nucleotide sequence include their regulatory regions.

## 5 Nonfunctionalisation: Are There Many “Pseudogenes” in Spider Genomes?

Apart from gene conservation, another rapid way for the organism to deal with the doubled amount of gene product in the cells immediately after gene duplication is preventing one of the gene copies from carrying out its function. This so‐called nonfunctionalisation (Rastogi and Liberles 2005), is thought of as a relatively quick response of the organism to counteract adverse effects of gene duplication. Because it restores the original meticulous regulation of the gene product (i.e. it restores the pre‐duplication status), nonfunctionalisation is generally expected to be the major fate of gene duplicates especially after a WGD (e.g. Lynch and Conery 2000), because this would mitigate the otherwise deleterious effects of the sudden duplication of gene product amounts in the cells. Thus, if the large number of duplicated genes in spiders is indeed caused by a WGD, one would at the same time expect to find a significant number of nonfunctionalised genes in the genome. Does this hold true for the genomes of spiders?

The nucleotide sequence of a genuine nonfunctionalised gene should change rapidly and randomly as compared to its conserved twin, because it is no longer subject to selection (reviewed in Prince and Pickett 2002; Rastogi and Liberles 2005). This provides a way to identify nonfunctionalised genes in the genome sequence by searching for genes that have diverged significantly from their more conserved twin. However, higher levels of sequence divergence can make a nonfunctionalised gene unrecognisable as a copy of another gene or even unrecognizable as a gene at all. In studies that focus solely on the analysis of the genome sequence, this would lead to an underestimation of nonfunctionalised gene copies in the genome sequence. The identification of nonfunctionalised copies of genes by computational annotation of genome sequences is thus challenging, and there has not yet been a systematic search for such genes in the available genome sequences of spiders.

Another way to identify nonfunctionalised genes is to study the gene products from genes and their significance for the function of the organism. There have been numerous large‐scale transcriptome studies in different spider species, but none of them has focused on systematically identifying genes with impaired RNA production that could point to possible “pseudogenisation”. No systematic genome‐wide studies of spatiotemporal gene expression patterns (e.g. via in situ hybridisation or protein detection using antibodies), of the effect of mRNA depletion (e.g. via RNAi) or of the effect of mutagenesis have been performed in spiders so far. Gene expression studies by whole mount in situ hybridisation of selected duplicated developmental genes indicate, that both duplicates are expressed (e.g. Aase‐Remedios et al. 2023; Janssen et al. 2021; Leite et al. 2018 for more examples please see references above), which would argue against nonfunctionalisation. There are, however, cursory reports in the literature, again from studies of a few duplicated developmental genes, that an altered phenotype is only observed after RNAi with one copy of the gene, but not with the other copy (Klementz et al. 2024; Pechmann et al. 2015). This could indicate that the second copy, although expressed, does not have a function in embryonic development. But the true reason for the failure to obtain RNAi phenotypes with certain duplicates of developmental genes is not known and has not been specifically investigated.

Taken together, there is currently no evidence for a significant number of nonfunctional genes in the genomes of spiders, which would argue against the presence of a WGD. However, the data basis for this assessment is very weak and is a by‐product of selective studies rather than being derived from dedicated systematic studies.

## 6 Subfunctionalisation and Specialisation: Are There Many “Complementing Gene Couples” In Spider Genomes?

Apart from either quickly eliminating the duplicated functionality (i.e. nonfunctionalisation) or fully accepting it (i.e. conservation), there are further possible outcomes after gene duplication. Subfunctionalisation like nonfunctionalisation is a way to reduce the amount of gene product after gene duplication towards pre‐duplication levels (Force et al. 1999; Lynch and Force 2000; Prince and Pickett 2002). In contrast to nonfunctionalisation, subfunctionalisation achieves the pre‐duplication status by affecting both gene copies at the same time, restricting the role of each of the duplicated genes. In other words, after subfunctionalisation both gene copies complement one another and together they perform the same functions as the single original gene. For example, the expression level regulation of each of the duplicates can be halved, so that the two copies produce the required normal amount of gene product together. Alternatively, the spatial or temporal regulation of the duplicates can be altered in a way that the two duplicates are never expressed in the same cells at the same time (i.e. complementary expression patterns). In this way, an expressing cell has both genes in its genome, but only one of the copies is expressed, which mimics the pre‐duplication state of the gene. Apart from alterations in the regulatory regions of the duplicated genes, the changes may instead affect the coding regions (e.g. Moriyama and Koshiba‐Takeuchi 2018). For example, an original gene coding for a protein with two functional domains A and B may be subfunctionalised into a copy which only codes for domain A and a second copy that only codes for domain B. In this case, the amount of gene product as such is still doubled, but the quantity of effective portions within the gene products are each halved and thus reduced to pre‐duplication levels.

So, long as the divergence of the two duplicates does not interrupt their ability to jointly perform the original pre‐duplication function, each copy may also evolve novel functions. This phenomenon is termed specialisation (He and Zhang 2005; Rastogi and Liberles 2005) and is usually classed as a separate category of gene duplication outcomes (see Figure 1).

Based on gene expression profiles, some gene pairs have been suggested to be subfunctionalised gene couples. For example, the Wnt‐family gene *wingless* (*wg*) has a conserved role in the development of the anterior portion of the body segments in arthropods. However, in spiders the *wg* gene is only expressed in a small part of the anterior portion of the segment (Damen 2002; Janssen et al. 2021, 2010). Other Wnt‐family genes are expressed in those portions of the segment, where *wg* is missing, and it has been suggested that in spiders several Wnt‐genes are required to form the continuous expression in the anterior portions of the segments known from other arthropods. Another example is the duplication of the Sox genes in spiders, from which all have been retained, and many seem to have undergone sub‐ or neofunctionalization (Bonatto Paese et al. 2018). Functional studies and expression pattern analysis comparing the expression of *Sox21b* in a harvestmen and a spider suggest an ancestral role of this gene in segmentation, which subfunctionalised in spiders after the duplication (Baudouin‐Gonzalez et al. 2021; Paese et al. 2018). Other studies have reported that for some gene pairs it is not possible to obtain a changed phenotype after RNAi with any of the two copies (Klementz et al. 2024; Pechmann et al. 2015). It has been speculated that in these cases RNAi treatment of the single genes has no effect, because the two copies complement or even compensate for each other. Neither the few observed cases of complementary gene expression patterns nor the cases of missing RNAi effect, however, have been further investigated and it is therefore unclear whether the two genes really complement each other functionally. Only in the study of Toll duplicates (Benton et al. 2016) there is compelling functional evidence that both copies have complementary functions and are thus genuine subfunctionalised copies.

All cases of subfunctionalisation and specialisation require the concerted evolution of the gene sequences of both duplicates rather than the unaltered retention of both copies (i.e. conservation) or the disuse of one copy (i.e. nonfunctionalisation). Significant divergence by sequence evolution of both duplicates makes it more difficult to identify such gene pairs based on sequence analysis alone. For example, in contrast to nonfunctionalisation there is no “conserved copy” that can serve as a signpost for gene identity and duplicate identification in the genome sequence. And it is challenging to glean from nucleotide sequence alone, whether the genes of a gene pair are functionally complementing each other; even if pairs of gene duplicates can be computationally predicted, there are currently no methods in spiders to also confidently predict that they are functionally complementary. It is therefore currently unclear, whether subfunctionalisation or specialisation have occurred in a significant number of the many duplicated genes in spiders.

## 7 Neofunctionalisation: What to Look for in Spider Genomes?

**Figure 4:**
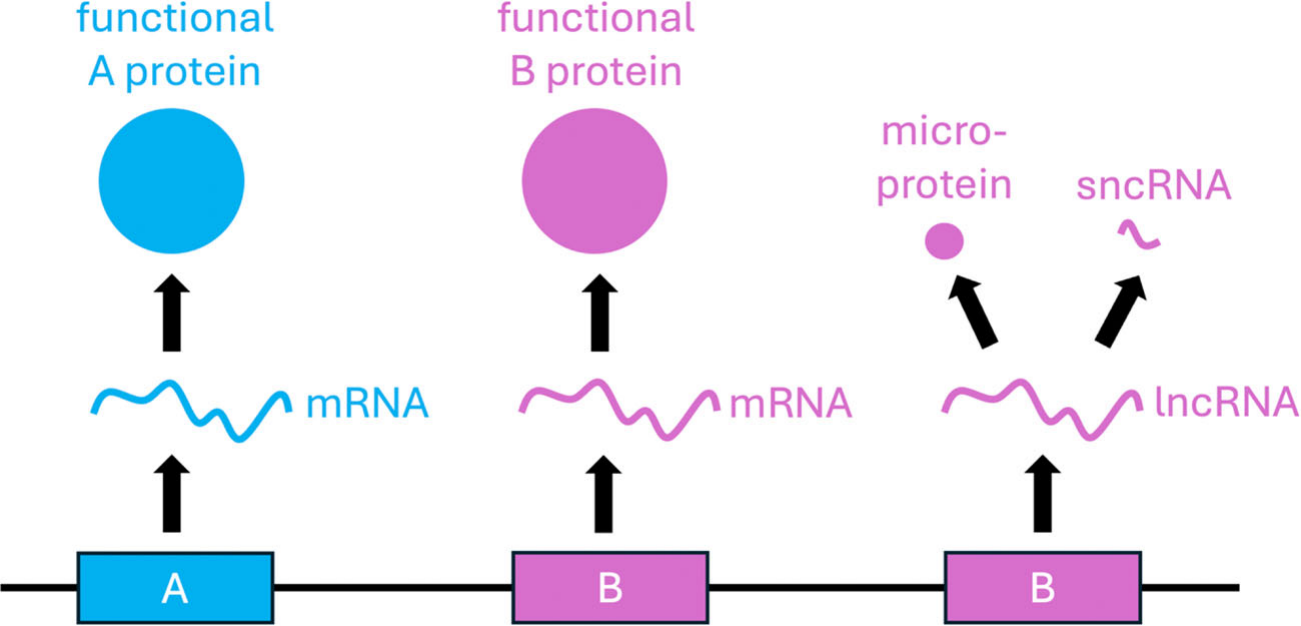
Complex facets of neofunctionalisation. Left: one gene copy (denoted as "A") retains the original gene function: in the example it produces mRNA that is translated into A protein. Centre: the neofunctionalised gene copy (denoted as "B") still produces mRNA and protein but has a changed regulatory and/or coding region, so that either the encoded B protein is structurally different from A protein, or B protein is expressed in a tissue or at a timepoint different from A protein. Right: alternatively, the neofunctionalised gene copy does not encode for a protein anymore; the transcribed RNA serves instead as a long noncoding RNA (lncRNA). Small open reading frames in lncRNA are translated into functional microproteins (Leong et al. 2022), or the lncRNA is processed into shorter RNA pieces (small noncoding RNA, sncRNA) that serve as versatile regulatory factors in the regulation of other genes (e. g. microRNA and small interfering RNA (siRNA)).

The basic principle of neofunctionalisation is that one copy of a gene retains the original function, and the other copy assumes a new function in the cell (summarized in DeGiorgio and Assis 2021). In practice, however, there are multiple facets of obtaining a new function (Figure 4). Changes may affect the regulatory or the coding region of the duplicate gene (summarized in Moriyama and Koshiba‐Takeuchi 2018). Minor such changes, e.g. the duplicate only gains a new sequence motif in its coding sequence, or it gains new regulatory elements that lead to expression in previously non‐expressing cells, but its coding sequence remains virtually the same, should still enable the discovery of such gene pairs by genome sequence analyses.

However, if the changes are more profound (e. g. Fig 4, right), it could be virtually impossible to recognize a gene as a copy of another gene, even if the other copy remains virtually unchanged.

Because the current methods of genome annotation largely rely on identifying genes by the features of their coding sequence, genes that change their coding region very strongly, or change their function from producing messenger‐RNA (mRNA) to producing noncoding RNA (ncRNA) will be missing entirely from the analysis, although these types of neofunctionalisation may contribute significantly to novel protein functions and novel gene regulation.

In all cases, if such strongly derived duplicates are nevertheless identified, they will likely be categorized as nonfunctionalisations, because they encode unusual proteins or no protein at all. Thus, sequence‐based genome analyses will underestimate the number of neofunctionalisations and overestimate the number of nonfunctionalisations.

Several previous studies of selected developmental gene pairs in spiders have postulated neofunctionalisation of one of the gene copies (Janeschik et al. 2022b; Turetzek et al. 2017, 2016). Subsequent studies, however, have not been able to support these conclusions and most of the cases of alleged neofunctionalisation are currently considered inconclusive (Klementz et al. 2024; Sharma 2023; Sharma and Gavish‐Regev 2024). The difficulties to conclusively identify neofunctionalisations in spiders are two‐fold. First, there are no clear criteria how to identify neofunctionalisations based on genome sequence analysis. Even though duplicated gene pairs can be computationally predicted by orthology analysis of genome sequences, there is currently no reliable method to differentiate computationally between subfunctionalisation, specialisation and neofunctionalisation for a given gene pair. Second, functional studies of gene pairs must adhere to high standards, to make a strong case for neofunctionalisation (Sharma 2023): the function of both genes must be known individually and in combination, and the function of the orthologous gene before duplication must be known. Appropriate methodology and appropriate outgroup model systems are not always available, and therefore, those standards are not met by most studies of the subject. Two examples from the Atlantic salmon genome (Lien et al. 2016) and the spotted gar genome (Braasch et al. 2016) showed that even if gene expression data, gene orthology and high quality level genomes are available, the identification of the fate of duplicated genes can produce variable results if different duplicate analysis approaches are used (Sandve et al. 2018). Many aspects such as clear definitions and criteria i.e. ranking the duplicates, integrating methods from phylogenetic analysis considering that selection does not affect all genes uniformly, as well as comparing gene expression divergence data with specifically defined models testing different duplicate fates and against neutral evolution are not yet available, but would be required to categorize duplicated genes correctly (summarized in Sandve et al. 2018).

In summary, although spiders possess pervasive gene duplications, feature a plethora of unique phenotypic novelties, and have undergone a massive diversification to over 52,000 extant species, there is currently no evidence for pervasive neofunctionalisation of duplicated genes to be the cause of these phenomena.

## 8 Only Two Different Evolutionary Trajectories After Gene Duplication?

It has been argued previously that the current view of the major evolutionary trajectories after gene duplication (see Figure 1) may involve too many rigid categories that are blurring the view for the actual processes after gene duplication (Sharma 2023). The focus on unflexible categories like neofunctionalisation or subfunctionalisation may not adequately reflect the dynamics of body plan evolution in chelicerates as well as in other metazoans.

Apart from the case where doubled gene action is beneficial as such, the main force during the initial stages after gene duplication is apparently the restoration of the pre‐duplication state with respect to the original gene function. All other processes that may take place simultaneously or consecutively are initially irrelevant, as long as they do not interfere with the restoration of the pre‐duplication state. Thus, there seem to be only two fundamental evolutionary processes after gene duplication:

1. Both copies diverge simultaneously from the original function while incessantly dividing the original function between them.
2. Only one copy diverges from the original function, thus relegating the original function to the other copy entirely.

In the first case, the outcome are two complementary copies that are mutually dependent, because both are required to perform the original function. This is a significant restriction because it limits sequence evolution to concerted changes in both genes. This does not, however, imply that the two genes cannot diverge significantly. It rather is reminiscent of coevolution of two species. Coevolution of species implies that the two species exert strong selection pressure on each other; it is unclear whether this concept can be transferred to the coevolution of duplicated gene pairs, but it is of note that coevolution of species can lead to strong mutualism, similar to what is expected in subfunctionalised or specialised gene pairs.

**Figure 5:**
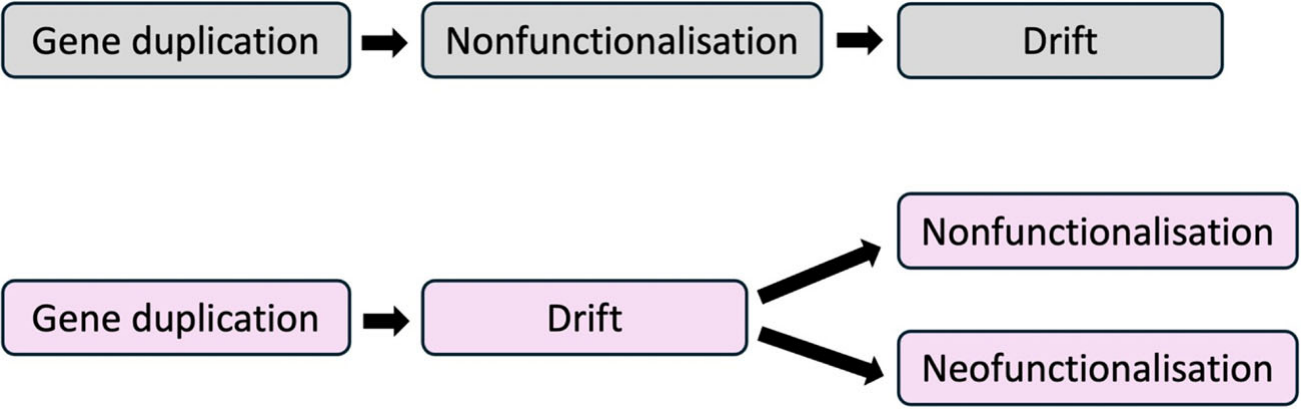
Conditional drift as the common initial state before non‐ and neofunctionalisation. Top: usually, nonfunctionalisation is thought to be the prerequisite for subsequent sequence evolution by drift, that finally leads to gene loss. Bottom: by contrast, a functionally redundant gene copy can enter conditional drift directly after gene duplication, the conditions being that it does not execute its previous (redundant) function and that it does not execute a deleterious function. All other neutral or beneficial functions are allowed, thus, conditional drift may endure or lead to nonfunctionalisation or neofunctionalisation.

In the second case, only one gene copy conserves the original gene function. The redundant function of the second gene copy is removed by sequence evolution. It is interesting to note, that “removal of the redundant function” only means that the gene is not allowed to execute its initial function, and that the gene is not allowed to execute a function that is deleterious for the organism. Apart from these two constraints, the gene is not functionally restricted and can be viewed as entering a state of conditional drift, as already proposed by (Lynch and Conery 2000) who have identified a period of relaxed selection, and evolution in a neutral manner early in the history of duplicate genes. During conditional drift the gene can be fully expressed and can also perform neutral functions in the cell. A recent study suggests that this process leads to nonfunctionalisation and gene loss as the default fate for gene duplicates, but it is not ruled out that a certain proportion of duplicate genes may also undergo neofunctionalisation or continued drift (Johri et al. 2022). Consequently, drift is not the result of nonfunctionalisation, but drift is a major state many gene duplicates enter long before they eventually are nonfunctionalised and lost or receive a new function (Figure 5).

Intriguingly, Johri et al. (2022) have demonstrated that in contrast to previous opinion (reviewed e.g. in (Innan and Kondrashov 2010)) the phase of neutral evolution of duplicate genes may last for long times. During this time, both copies are retained in the genome, and such continued retention of gene duplicates may then erroneously be attributed to neofunctionalisation. Thus, it could be possible that many duplicated spider gene pairs are currently in this prolonged phase before gene loss that has been identified by Johri et al. (2022). Their retention and even their differential expression is therefore not an indication of neofunctionalisation but simply reflects their current state of drift. This line of reasoning may also explain the lack of clear cases of nonfunctionalisation and neofunctionalisation in spiders as reviewed above: the genes have simply not yet reached these phases that would complete their drift.

This, however, does not mean that no cases of neofunctionalisation have occurred in spiders. One fascinating case has recently been identified: the duplicate of an *Iroquois*‐related gene, named *waistless*, has acquired a new function in a spider‐specific phenotypic novelty, namely the spider pedicel, which is the elongated and thin first segment of the opisthosoma (abdomen) (Setton et al. 2024). What makes this example even more intriguing and insightful is the predicted age of this neofunctionalised duplicate: the duplicate is much older than the entire group of the Chelicerata. First, this implies that the duplicate gene must have been drifting for a very long time before finding a novel function in controlling a spider‐specific morphology. Second, and more importantly, this case of neofunctionalisation, although clearly involved in a spider‐specific phenotypic novelty, is not linked to any chelicerate WGD event and raises the question of how important the possible arachnopulmonate WGD event was for the evolution of phenotypic novelty in spiders.

A second case for neofunctionalisation in spiders has recently been identified in a duplicate of a GATA‐related gene, named *fuchi nashi* (Iwasaki‐Yokozawa et al. 2022). There are two fascinating aspects about this case. First, again the age of the duplication event is surprising: it occurred in a subgroup of the Araneae and thus is present only in a smaller fraction of spider diversity and is not related to WGD events. Second, despite this very young age of the gene, it has a key function in the processes of zygotic gene activation (at the transition from maternal transcripts to zygotic transcription) and of endoderm specification during early embryonic development. Intriguingly, these processes are fundamental processes in all spiders, not only in the subgroup that possesses *fuchi nashi*. Thus, these functions must have been performed by another gene or by several other genes before they have been taken over by *fuchi nashi*. In this case, the neofunctionalisation of *fuchi nashi* is only partially neofunctionalisation in the full sense of the term: it represents the gain of a new function for the duplicate gene (in the sense that the gene did not have this function before), but the function itself is not new: it has been performed by other genes before the origin of *fuchi nashi*. This variety of neofunctionalisation, termed here “usurpation”, where a gene duplicate intrudes a genetic network that is much older than itself and seizes a function at or near the top of that network, may in fact be an outcome that is more frequent than genuine neofunctionalisation. It is of note, that some well‐studied duplicates in metazoan animals show usurpation.

For example, the trithorax‐like duplicate *lolal* originated in the mandibulate arthropods, but is a key player in patterning the dorsoventral axis, which is present in all bilaterian animals, not just mandibulate arthropods (Quijano et al. 2016). Another example is the transcription factor *duxbl*, which is a duplicate of *dux* and originated in the lineage of murine mammals (mice and rats) but is critically involved in the control of the exit from totipotency, which is a fundamental step in the early embryogenesis of all mammals, not just mice and rats (Vega‐Sendino et al. 2024). Arguably, the most famous example for a recent duplicate that usurped a much older function at a key position in a genetic network, is the Hox gene duplicate *bicoid* (Stauber et al. 1999). The *bicoid* gene originated in the lineage of dipteran insects and is the main player in anterior patterning and head development in flies, although the development of an anterior end with a head is a general feature of animals. Thus, usurpation rather than genuine neofunctionalisation may be a widespread fate of drifting duplicate genes to escape the default fate of nonfunctionalisation and gene loss.

## 9 Conclusions

In comparison to well‐studied WGD events in vertebrates, large‐scale duplications in other animals seem to be less prominent or were not as intensively studied due to the lack of high‐quality genome sequences. With advances in sequencing technologies, the number of available high‐quality genome sequences is increasing and expansion of gene repertoire by duplication is found in more and more species, including chelicerates, a group of arthropods. Most studies currently focus to reveal if the large‐scale duplications in e.g. horseshoe crabs, spiders, and scorpions are derived from one or several ancient WGD events. While the generation of more high‐quality genome sequences from phylogenetically relevant positions across the chelicerate tree combined with orthology and macrosynteny analyses have exciting potential to shed light on the evolution of genomes, it most likely will not provide straightforward answers to further important questions. In the future it would be interesting to systematically identify and study genome duplication events focusing on the regulation and function of the gene duplicates, in addition to sequence similarity and genome evolution.
